# Soil Salinity Drives the Distribution Patterns and Ecological Functions of Fungi in Saline-Alkali Land in the Yellow River Delta, China

**DOI:** 10.3389/fmicb.2020.594284

**Published:** 2020-12-23

**Authors:** Chao Yang, Juan Sun

**Affiliations:** Grassland Agri-Husbandry Research Center, College of Grassland Science, Qingdao Agricultural University, Qingdao, China

**Keywords:** soil salinity, soil pH, fungal community and diversity, salt tolerant fungi, yellow river delta

## Abstract

High soil salinity is the main factor that limits soil microbial activity in the Yellow River Delta (YRD); however, its effects on fungal community and ecological function are unknown. Here, we comparatively investigated the diversity and structures of soil fungal communities targeting the internally transcribed fungal spacer gene using Illumina MiSeq sequencing methods under a salt gradient with five levels, namely, Low: low-salinity soil, Medium: medium-salinity soil, High: high-salinity soil, Extreme: extreme-salinity soil, and a non-salted site as the control (Non-saline). The results show that bulk density (BD) values significantly increased (*p* < 0.05), while significantly lower values of soil total carbon (TC), total nitrogen (TN), and fungal Shannon and Chao indexes were observed as the salinization gradient increased (*p* < 0.05). The relatively high levels of the families *Nectriaceae* and *Cladosporiaceae* distinguished two of the clusters, indicating two enterotypes of low (Non-saline and Low) and high (Medium, High, and Extreme) salinity soils, respectively. The family *Nectriaceae* was most abundant in the networks, and the positive correlations were more pronounced than negative correlations; however, *Cladosporiaceae* was the family most negatively correlated with others based on the network analysis. At the ecological function level, plant saprotrophs and litter saprotroph were significantly less abundant in extremely saline soil than non-saline soil. The change in soil properties (TC, TN, and BD) caused by soil salinization [salt and electrical conductivity (EC)] regulated the diversity of soil fungal communities, and ecological function, as indicated by Pearson correlation analyses. We suggest further investigation into the ecological functions of soil microorganisms in the extremely saline-alkaline soils of the YRD.

## Introduction

Salinization is one of the main problems causing land degradation and crop yield reduction throughout the world (Rath and Rousk, 2015). In the Yellow River Delta (YRD) in China, soil salinization has spread at an unprecedented rate from coastline to inland areas over the past two decades (Zhao et al., 2020) due to sea-level rise and increased groundwater abstraction (Cubasch et al., 2014). High saline-alkali soils are the essential factor that not only negatively influences vegetation growth (Cui et al., 2009) but also affects soil respiration, soil microbial biomass, and the microbial growth rate (Campbell and Kirchman, 2013; Rath et al., 2019a). Hence, it is important to evaluate the effect of soil salinization on soil microbial community structure for the improvement of saline-alkali lands in the YRD.

Soil salinity has been shown to be the most important factor affecting the global distribution of soil microorganisms (Lozupone and Knight, 2007; Auguet et al., 2010). Salinity is a major factor shaping soil bacterial diversity and composition in many natural habitats (Campbell and Kirchman, 2013; Zhao et al., 2020). For this reason, a salinity gradient is likely to affect soil fungal patterns (Mohamed and Martiny, 2011). In the complex soil ecosystem, fungal diversity has important consequences for ecosystem functions (van der Heijden et al., 2008). For example, mycorrhizal fungi increases nutrient capture by expanding the surface area of plant roots (Kramer et al., 2012). Saprotrophic fungi are involved in organic matter decomposition, and greater saprotrophic fungal diversity increases organic matter decomposition (van der Wal et al., 2013; Schmidt et al., 2019). Pathotrophic fungi affect crop growth, but they also control other plant or fungal pest populations (Vega et al., 2009; Wang and Wang, 2017). Despite their importance to ecosystems, few studies have considered how salinity affects the ecological function of fungi. In particular, the fungal structure and function at different salinities and pH values in the YRD have not been investigated.

It is well known that environmental factors have significant effects on soil fungal communities (Bachelot et al., 2016). For example, soil pH is one of the most important factors affecting soil fungal communities (Geml et al., 2014; Hu et al., 2017), and Maestre et al. (2015) revealed that soil pH was negatively related to fungal diversity at the global scale. Additionally, Geml et al. (2014) observed that soil fungal communities were closely related to the soil carbon (C) and nitrogen (N) contents. In saline-alkali soils of temperate grassland in northern China, our findings suggest that soil pH was negatively correlated with fungal diversity compared with soil salinity and the C/N ratio (soil carbon to nitrogen ratio; Yang et al., 2020). As soil salinization and alkalization frequently co-occur, it is necessary to identify which factor has the greater influence on the composition and diversity of soil fungi in the severe salinization region of the YRD. Groups of fungi with different ecological functions had different relationships with soil properties (Schmidt et al., 2019). For instance, the relative proportion of mycorrhizal fungi was negatively correlated with soil pH whereas animal pathogens were positively correlated with soil organic matter in cropland (Schmidt et al., 2019). Far less is known about how those groups of fungi respond to the variation in soil properties caused by soil salinity.

Although the microbial responses to salinity in saline-alkali lands have become a hot issue (Hu et al., 2016), shifts in structure and function of fungi as determined using soil fungal internally transcribed spacer methods in saline-alkali soils of the YRD have rarely been reported. Traditionally, almost all biodiversity studies of fungal ecology only consider species composition and disregard the interactions among different fungi; however, network interactions could be important to ecosystem processes and functions than species diversity (Zhou et al., 2011). In this study, we measured the soil fungal composition and assessed networks of co-occurrence using high-throughput sequencing technology along a salinity gradient, and we also evaluated the effect of salinity on the ecological function of fungi using FUNGuild software (Nguyen et al., 2016). The aims of the present study were (1) to identify the community composition and the fungal diversity along the salinity gradient, (2) to determine the co-occurrence networks among soil fungi and identify their ecological functions in saline soils, and (3) to evaluate the key soil factors affecting the soil fungal community structure and ecological function.

## Materials and Methods

### Study Sites and Soil Collection

**Figure 1:**
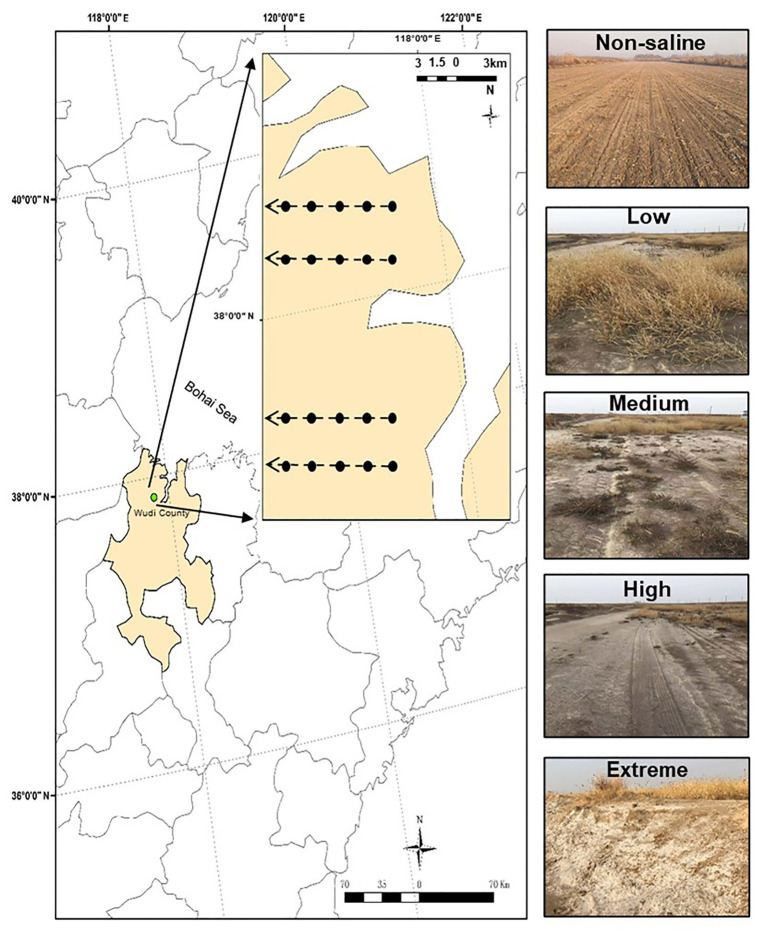
The study region and five salinity gradients with different EC values. The map was created with ArcGIS v 10.2 (http://www.esri.com/arcgis/about-arcgis). Non-saline: non-salted, Low: low-salinity soil, Medium: medium-salinity soil, High: high-salinity soil, and Extreme: extreme-salinity soil.

The sampling area was a part of the YRD in northern Shandong on the southern shore of the Bohai Sea (37°54'60''N, 117°57'33''E, elevation 1 m). This area has a semi-humid continental climate characterized by a mean annual air temperature and rainfall of 12°C and 600 mm, respectively. The site has a coastal saline soil with a silt-sand texture. We selected five salinity levels from low to extreme salinization (Figure 1). In brief, land dominated by *Setaria viridis* and low-salt tolerant vegetation was selected as low-salinity soil (Low). *Suaeda salsa* and medium-salt tolerant vegetation dominated saline-alkali lands that were selected as medium-salinity soil (Medium). Saline-alkali lands without vegetation growth were selected as high-salinity soil (High), and Extreme-salinity soil (Extreme) was saline-alkali lands with salt crystallization. Maize (*Zea mays*) croplands with low salinity were selected as the control (Non-saline), and these are mainly affected by flooding freshwater. The mean soil electrical conductivity (EC) value ranged from 0.92 (Non-saline) to 1.78 ds/m (Low), to 3.16 ds/m (Medium), to 17.26 ds/m (High), and finally to 34.41 ds/m (Extreme; Table 1).

**Table 1:** One-way ANOVA of the soil properties of non-salted (Non-saline), low-salinity soil (Low), medium-salinity soil (Medium), high-salinity soil (High), and extreme-salinity soil (Extreme) sites. Values are mean ± SE.

	EC (ds m^−1^)	Salt (%)	pH	BD (g cm^−3^)	TC (g kg^−1^)	TN (g kg^−1^)	C/N ratio
Non-saline	0.92(0.10)e	0.04(0.01)d	8.70(0.05)a	0.96(0.02)d	21.10(1.13)a	0.63(0.05)b	34.70(4.59)d
Low	1.78(0.37)d	0.16(0.05)c	8.18(0.07)c	1.07(0.02)c	17.40(0.50)b	0.85(0.09)a	21.11(2.12)e
Medium	3.16(0.23)c	0.18(0.01)c	8.75(0.04)a	1.23(0.02)b	16.35(1.17)b	0.40(0.04)c	41.59(3.20c)
High	17.26(1.01)b	0.88(0.09)b	8.56(0.01)b	1.32(0.01)a	14.33(0.50)c	0.38(0.08)c	47.96(1.69)b
Extreme	34.41(0.63)a	3.58(0.13)a	8.49(0.01)b	1.34(0.01)a	10.78(0.11)d	0.20(0.01)d	53.88(0.54)a

EC, electrical conductivity; BD, bulk density; TC, soil total carbon; TN, soil total nitrogen; and C/N ratio, soil carbon to nitrogen ratios. In the list, different lowercase letters indicate the significant relationships (p < 0.05) among the five salinity gradients using the Duncan’s test.

Four transects with a distance of about 3 km represented four repetitions, and five plots (Non-saline, Low, Medium, High, and Extreme) separated at least 500 m from each other were randomly established along each transect (Figure 1). To remove plant root disturbance, the bulk soil (0–15 cm) was collected using a five-spot sampling method in each plot (5 × 5 m^2^) in October 2019, and we mixed the samples into one composite sample. In total, 20 samples (five salinity levels × four repetitions) were collected in plastic bags, and the samples were carefully sieved through a 2 mm mesh. Then, we divided the soil samples into two subsamples. One subsample was air-dried for the analysis of soil basic properties, and the other part was stored in a −80°C freezer for microbiological analysis. Soil total carbon (TC) and total nitrogen (TN) concentrations were measured using a CHNS Element Analyzer (Elementar, Germany). Soil pH and EC were measured using a glass electrode in a 1:5 soil: water suspension. Soil bulk density (BD) was calculated using the ring knife method at a 0–15 cm depth. In brief, a foil sampler with a volume of 100 cm^−3^ was used to obtain the samples, which were then dried at 105°C for 24 h. The soil salt content was determined in a mixture with a soil: water ratio of 1:5, and the soil extract was then dried at 105°C for 24 h (Yang et al., 2020).

### Fungal DNA Extraction and ITS Gene Sequencing Amplification

The fungal extraction and determination methods refer to our previous research (Li and Yang, 2019). In brief, the genomic DNA was extracted from each soil sample using a FastDNA®SPIN Kit for soil (MP Biomedicals, CA, United States). We accurately weighed 0.30 g soil sample from each treatment. Soil DNA integrity was then detected by 0.8% agarose gel electrophoresis. The non-coding region of fungal internally transcribed spacer (ITS) was amplified using ITS1 (5'-CTTGGTCATTTAGAGGAAGTAA-3') and ITS2 (5'-GCTGCGTTCTTCATCGATGC-3') primers (White et al., 1990). The PCR analysis included pre-denaturation at 95°C for 3 min; 27 cycles at 95°C for 30 s, annealing at 55°C for 30 s, elongation at 72°C for 45 s, and an extension at 72°C for 10 min.

Illumina MiSeq sequencing produced double-ended sequence data (2 × 300) according to standard protocols performed by MajorBio Bio-Pharm Technology Co. Ltd. (Shanghai, China). The obtained sequences were first filtered using the quantitative insights into microbial ecology. Raw FASTQ files were de-multiplexed and quality-filtered with the following criteria: (i) 300-bp reads were truncated at any site with an average quality score <20 over a 50-bp sliding window, and truncated reads shorter than 50 bp were discarded; (ii) exact barcode matching, less than two nucleotide mismatches in the primer, and no ambiguous characters in the read; and (iii) only overlapping sequences longer than 10 bp were assembled according to their overlapped sequence. UCLUST was used to sort the unique sequence set as an operational taxonomic unit with a 97% identity threshold.

### Statistical Analysis

One-way ANOVA was used to identify the soil fungal Shannon diversity index, fungal Chao 1 richness index, and soil physicochemical properties of the five salinization levels. The level of significance was defined at *p* < 0.05 using Duncan’s test in SPSS (ver. 19.0). Nonmetric multidimensional scaling (NMDS) analysis based on Bray-Curtis similarity matrices was performed to identify the total structural changes in soil fungi, and significance was tested by analysis of similarities (ANOSIM) in PAST (ver. 3.25). We calculated the Jensen Shannon distance (JSD) according to the abundance of fungi at the family level, and the maximization of the Calinski–Harabasz (CH) index was performed to select the optimal number of clusters using the *k*-medoids algorithm (PAM clustering) with R statistical software (ver. 3.6.3) using seven dissimilarity metrics (Tyakht et al., 2013). Redundancy analysis (RDA) was performed to analyze the relationship between the soil physicochemical properties and the whole fungal communities in terms of abundance at the family level. The significance of the effect of each variable was examined using Mantel tests (permutations = 999), and the resulting significance level was tested by the Mantel r statistic and *p* values. We used the Networkx software to establish the co-occurrence networks between families. The networks were constructed by calculating the correlation between families (coefficient was >0.5 and *p* was <0.01), and we evaluated the correlation information among families according to the transitivity, diameter, average shortest path length, degree and clustering of the networks. Using fungal ITS sequence data, we conducted both a phylogenetic and functional group analysis based on FunGuild (Nguyen et al., 2016) to assign fungal taxa into three nutrition modes – saprotrophy, symbiotrophy, and pathotrophy. The correlations between soil property parameters and the abundances and function of fungi were assessed by Pearson analyses in PAST (ver. 3.25).

## Results

### Soil Physiochemical Property Responses to Different Salinity Levels

The soil salt, EC, and BD values significantly increased, while significantly lower values of TC were observed as the salinization level increased (Table 1; *p* < 0.05). In particular, soil pH was highest in medium salinity soil (*p* < 0.05). Soil TN decreased by 25.9, 52.9, 55.3, and 76.5% in non-saline, medium, high, and extreme salinity soils, respectively, compared with that in low salinity soil (*p* < 0.05). Non-saline, medium, high, and extreme salinity soils exhibited an increased soil C/N ratio of 0.64, 0.97, 1.27, and 1.55 times, respectively, compared with that in low salinity (*p* < 0.05).

### Responses of Fungal Communities and Functions to Soil Salinity

**Figure 2:**
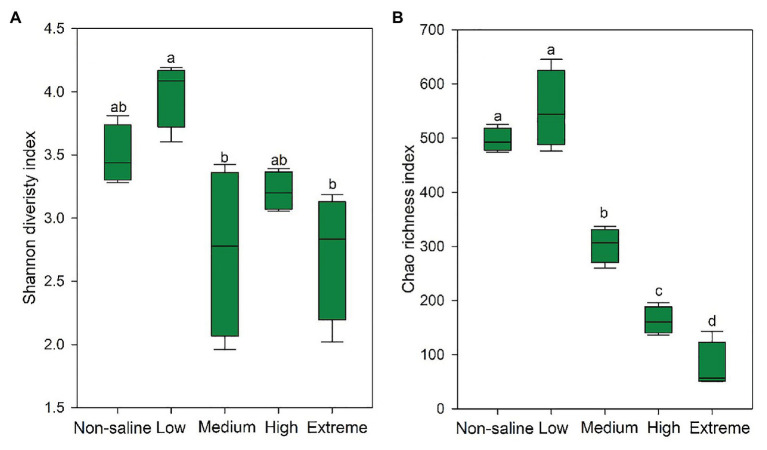
The Shannon diversity **(A)** and Chao richness **(B)** indexes of the soil fungi in five different salinized soils, and the significant relationships at *p* < 0.05 were indicated by different letters using the Duncan’s test.

**Figure 3:**
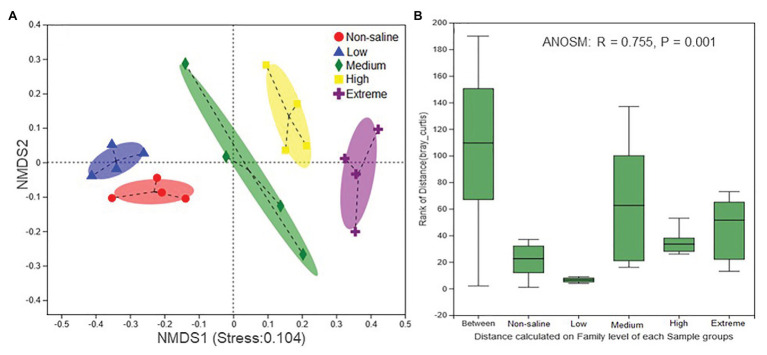
The nonmetric multidimensional scaling (NMDS) ordinations based on the relative abundance of the fungal communities in five different salinized soils **(A)** and the significant differences between the community structures of each salinity level were evaluated using an analysis of similarities (ANOSIM; **B**).

**Figure 4:**
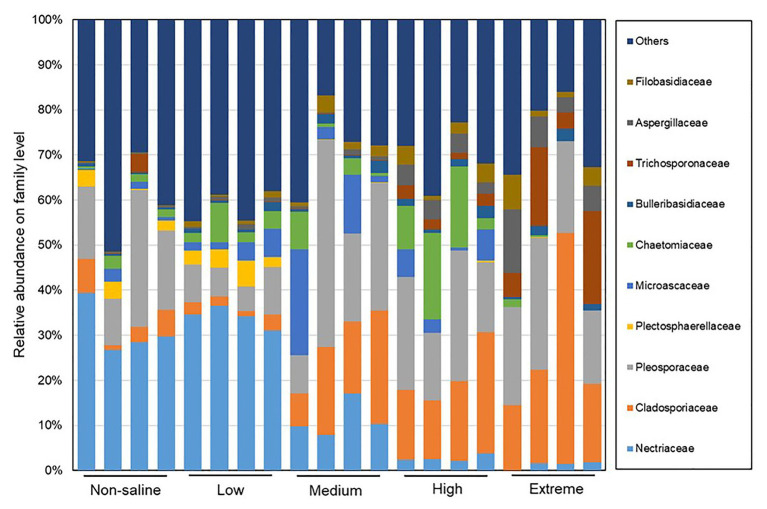
The distribution patterns of the soil fungal family compositions in five different salinized soils.

**Figure 5:**
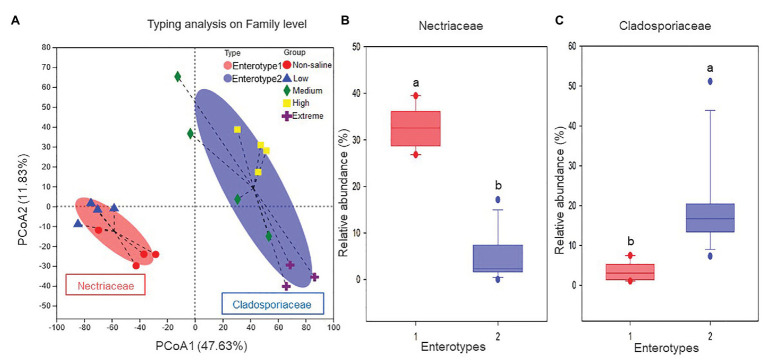
The enterotype analysis for five different salinized soils: **(A)** the clustering on the first two principal components, and the proportions of fungal taxa characteristic of **(B)** *Nectriaceae* and **(C)** *Cladosporiaceae*.

**Figure 6:**
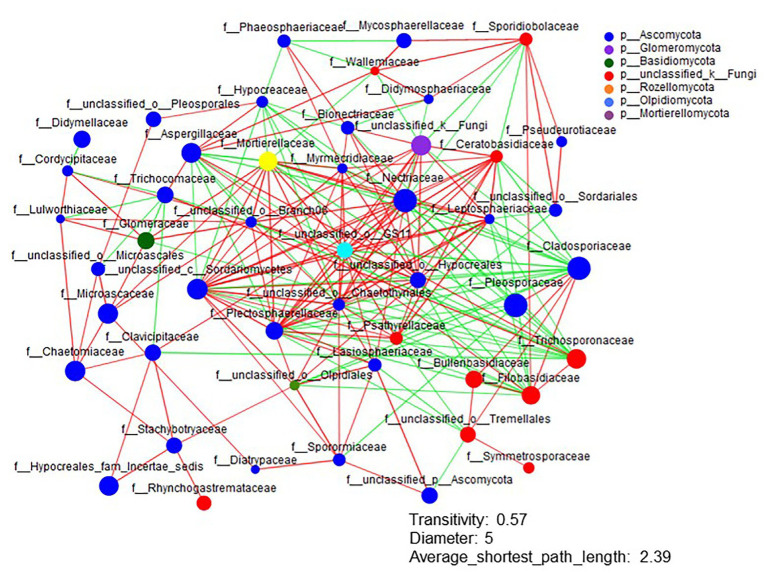
The network analysis of the fungal interactions from non-saline soils to extreme salinity soils. Colors of nodes represent different major phyla, and the node representing the family is shown inside. A red line indicates a positive interaction (coefficient was >0.5 and *p* was <0.01), whereas a green line indicates a negative interaction (coefficient was <−0.5 and *p* was <0.01) between two individual nodes.

**Figure 7:**
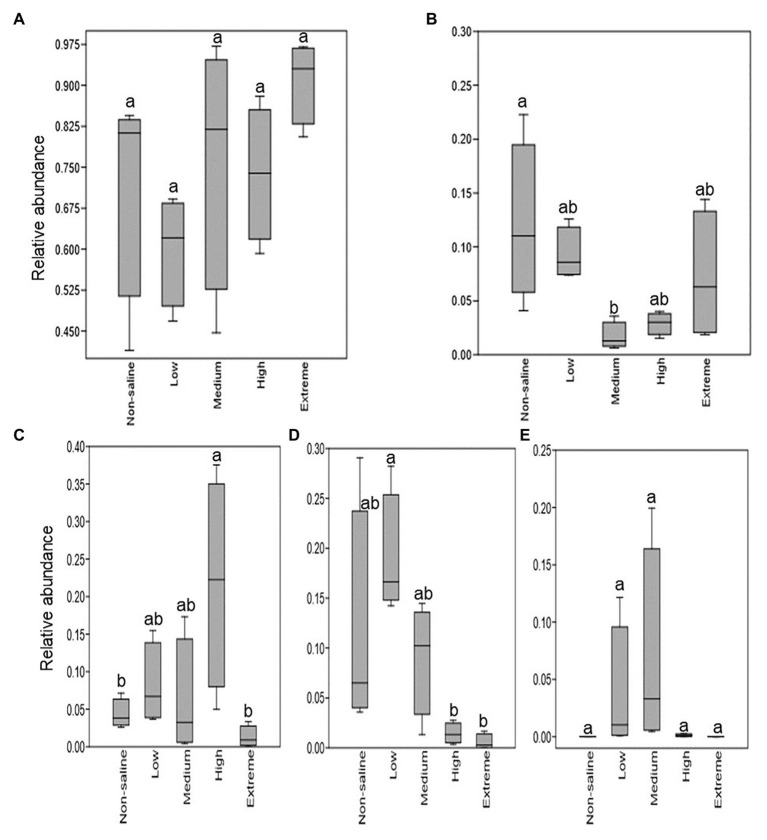
The relative fraction of fungal guilds that show significant differences with five different salinized soils. **(A)** Animal pathogens; **(B)** plant pathogens; **(C)** plant saprotrophs; **(D)** litter saprotrophs; and **(E)** arbuscular mycorrhizal. Different letters indicate statistically significant differences between gradients (*p* < 0.05).

The Shannon diversity of soil fungi in extreme salinity soil was significantly lower than that in low salinity soil (Figure 2A). In addition, significantly lower values of the soil fungal Chao richness index were observed in extremely saline soil (Figure 2B; *p* < 0.05). The NMDS and ANOSIM tests are shown in Figures 3A,B. NMDS showed that the fungal compositions in low, medium, high, and extreme salinity soils significantly differed from those in non-saline soil (stress = 0.104), and ANOSIM further confirmed that the Bray–Curtis distance between soil samples was greater than that within soil samples (*R* = 0.755, *p* = 0.001). The *Nectriaceae* and *Plectosphaerellaceae* families were the main microflora in the non-saline and low salinity samples (Figure 4); however, soil salinization dramatically increased the relative abundance of *Cladosporiaceae* from non-saline to extreme salinity soil. Additionally, the CH index showed that the data naturally separated into two clusters based on the JSD method (Supplementary Figure S1), and non-saline and low salinity belonged to the *Nectriaceae* enterotype based on the CH index (Figure 5A), which was significantly higher than that in medium, high, and extreme salinity soils (Figure 5B). In contrast, medium, high, and extreme salinity soils belonged to the *Cladosporiaceae* enterotype based on the CH index (Figure 5A), which was significantly higher than that in non-saline and low salinity soil (Figure 5C). Our network results showed a high level of connectivity within the saline soils with transitivity, diameter, and average shortest path length was 0.57, 5, and 2.39, respectively (Figure 6). The degree of family *Nectriaceae* was highest in the networks (Supplementary Table S1), and the positive correlations were higher than negative correlations; however, *Cladosporiaceae* was the family most negatively correlated with others. The clustering of *Leptosphaeriaceae* was highest in the networks (Supplementary Table S1), indicating it is highly important in saline soils. At the ecological function level, plant pathogens had significantly lower numbers under medium salinity soils, and plant saprotrophs and litter saprotrophs were significantly lower in extremely saline soil than in non-saline soil. There were no significant differences in the numbers of animal pathogens and arbuscular mycorrhizae along the salinity gradient (Figure 7).

### Soil Properties With Different Salinities Regulate Fungal Diversity, Communities, and Functions

**Figure 8:**
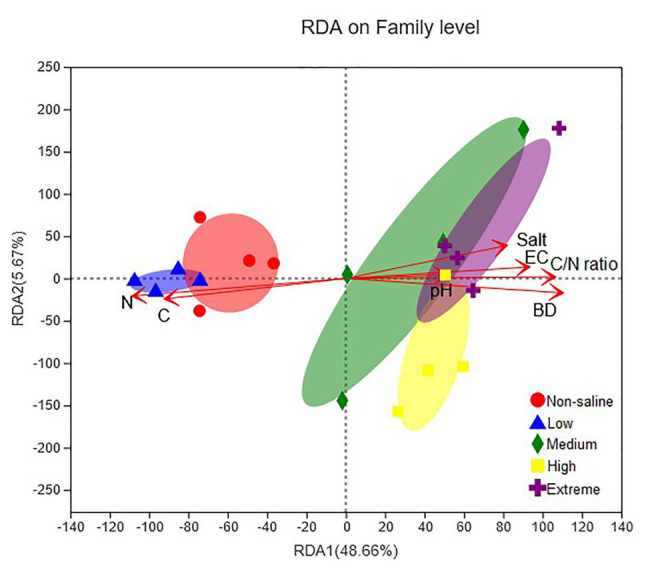
Redundancy analysis (RDA) showing the impact of soil physiochemical properties (pH, EC, salt content, BD, TC, TN, and C/N ratio) on the fungal community structure. The significance of the effect of each property was assessed using Partial Mantel tests (permutations = 999).

A combination of variables explained 59.46% of the variance of the fungal communities, shown in the RDA biplots (Figure 8). The Partial Mantel test showed that the soil EC (Mantel *r* = 0.61, *p* = 0.001), BD (Mantel *r* = 0.54, *p* = 0.001), and pH (Mantel *r* = 0.21, *p* = 0.021) were positively and negatively correlated with soil salt (Mantel *r* = 0.48, *p* = 0.001) and soil TC (Mantel *r* = 0.55, *p* = 0.001), respectively, which significantly influenced the fungal communities (Supplementary Table S2).

**Figure 9:**
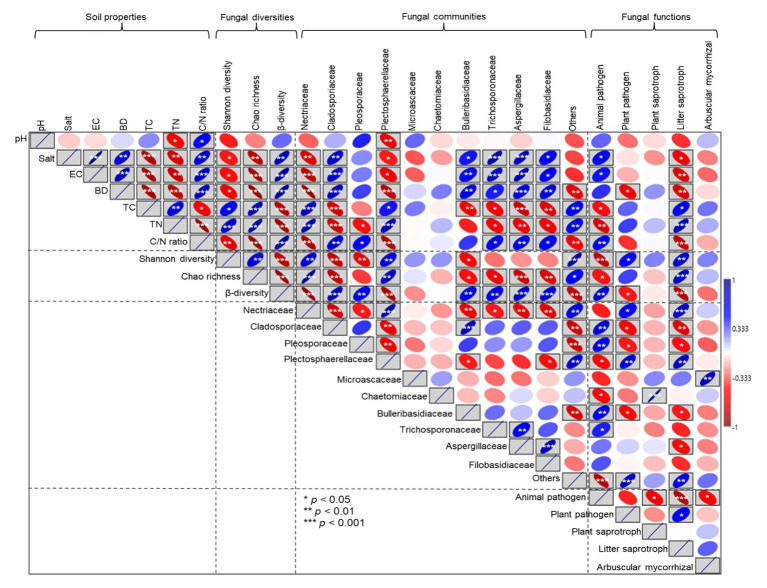
Pearson correlation analyses between soil property parameters and fungal diversity, the relative abundances of specific fungal families, and ecological functions. EC, electrical conductivity; TC, total carbon; TN, total nitrogen; and C/N (soil total carbon/nitrogen). The blue upward sloping ellipse indicates the positive correlation, whereas the red downward sloping ellipse indicates negative correlation. The width of the ellipse represents the level of correlation. *, **, and *** in the box indicate the significance along the paths at *p* < 0.05, *p* < 0.01, and *p* < 0.001 levels, respectively.

Pearson correlation analyses showed that soil salt, EC, and BD had a weakly significant negative correlation with Shannon diversity (*p* < 0.05). In contrast, an extremely significant negative correlation was observed between soil salt, EC, and BD and Chao richness (*p* < 0.001); however, soil salt, EC, and BD had an extremely significant positive correlation with β diversity (*p* < 0.001; Figure 9). Soil pH had a significant negative correlation with the relative abundance of *Plectosphaerellaceae* (*p* < 0.05). The relative abundances of *Nectriaceae* and *Plectosphaerellaceae* were decreased as soil salt, EC, BD, and the C/N ratio increased (*p* < 0.05). By contrast, the relative abundances of *Cladosporiaceae*, *Bulleribasidiaceae*, and *Aspergillaceae* were increased with increases in soil salt, EC, BD, and the C/N ratio (*p* < 0.05). The relative proportion of animal pathogens was positively correlated with salt and EC and negatively correlated with C and N concentrations, and plant pathogens were negatively correlated with soil BD (*p* < 0.05). There was a positive correlation between litter saprotrophs and soil C and N concentrations; however, litter saprotrophs were also negatively correlated with soil salt, EC, and BD (*p* < 0.05; Figure 9).

## Discussion

### Salinity Effect on Soil Properties, Fungal Communities, and Functions

Generally, the soil pH, salt content, and EC exhibit collinearity in saline-alkali soils (Zhao et al., 2018). Here, the soil salt content and EC significantly increased as the salinization level increased, which is in line with our previous study (Yang et al., 2020); however, soil pH showed no significant increase in the salinization level increased in this study, indicating the correlation between soil pH and salt is limited by the range of salt value, especially in the extremely saline soils of the YRD, China (Hu et al., 2016; Zhao et al., 2020). The negative relationship between soil TC and the salt content in the present study is consistent with the results reported by Morrissey et al. (2014) and is mainly due to the poor growth of plants affected by salinity, resulting in a low amount of organic carbon in the soil (Wong et al., 2010). Additionally, in the present study, the soil C/N ratio was higher in saline-alkali soils than in low salinity soils because the decrease in soil TN was higher than that in soil TC. As salinity increased, the soil BD increased significantly, which is consistent with the findings of Zhao et al. (2017), who reported that the soil BD increased along a salinity gradient in a drained coastal wetland, the YRD, China.

In agreement with other studies (Chowdhury et al., 2011; Elmajdoub and Marschner, 2015), salinity changed the microbial community structure because the difference in their tolerance to salinity. Our previous study found that Shannon diversity values in saline-alkali soils from grassland were significantly lower than those in low salinity soils (Yang et al., 2020), which was also confirmed in the present study. There was a weakly significant correlation between Shannon diversity and the soil salt content, and the similar salt content in low and medium salinity soils may not have been sufficient to cause detectable Shannon diversity in the present study. In addition, significantly lower values of the Chao richness index were observed in extreme salinity soil, which showed an extremely significant negative correlation with the soil salt content. Numerous studies have shown that fungal communities are influenced by soil salinity (Mohamed and Martiny, 2011; Krishnamoorthy et al., 2014). High soil salinity increased the relative abundance of the fungal phylum *Ascomycota* (Kim et al., 2019; Yang et al., 2020). Our results confirmed this: the fungal phylum *Ascomycota* and the related families *Cladosporiaceae* were significantly more abundant in high-saline-alkali soils, whereas, *Nectriaceae* and *Plectosphaerellaceae*, assigned to *Ascomycota*, were significantly less abundant in high saline-alkali soils, indicating an inconsistent response within the phylum *Ascomycota*, suggesting that shifts in community composition were mostly driven by shifts in soil salinity, with more salt-tolerant species (*Cladosporiaceae*) replacing less salt-tolerant ones (*Nectriaceae* and *Plectosphaerellaceae*; Rath et al., 2019b). It is difficult to explain those inconsistent responses, and there are few studies on the mechanism and function of salt tolerance of soil fungi. We here report that many species that form endospores and are thus able to survive in extreme environments and many of these species are known to be salt tolerant (Takami, 2011). We speculate that the ability to form spores might give *Cladosporiaceae* an advantage and allow them to survive the acute effects of salt exposure and grow more abundant after other fungi have died off. This must be studied in detail.

The recent report by Wang et al. (2019) indicated that the adjustment of microbial interactions could be a strategy by which microbes cope with intense salinity and alkalinity stresses. Generally, positive links imply cross feeding and niche overlap, while negative relationships represent competition in the network (Zheng et al., 2017). In the present study, more positive links in family *Nectriaceae* were observed in the co-occurrence networks between species; however, *Cladosporiaceae* was the family most negatively correlated with others, indicating their crucial roles in competition for nutrients, water, and dissolved oxygen under restricted resource conditions in saline alkaline soils (Wang et al., 2019). In soil ecosystems, fungi comprise various ecological guilds (Nguyen et al., 2016). We conducted a functional group analysis based on the recently developed open annotation tool FunGuild (Nguyen et al., 2016) to assign fungal taxa to three ecologically functional groups–saprotrophy, pathotrophy, and symbiotrophy. Saprotrophic fungi (e.g., plant and litter saprotrophs) grow throughout the soil–litter interface, serve as the primary agents of plant litter decomposition (Crowther et al., 2012). Specifically, plant saprotrophs and litter saprotrophs were significantly less abundant along a salinity gradient, which was conducive to the non-saline (*Zea mays*) and low salinity soils (*Setaria viridis*) with high plant and litter biomass. Thus, we inferred that constantly reduced saprotrophic fungi in saline soils would decrease the rate of decomposition of plant litter and old soil C, which would eventually affect soil organic C and N turnover and accumulation (Yang et al., 2017). However, no significant differences in the numbers of animal pathogens were observed along a naturally inhomogeneous salinity gradient in this study. The decrease in soil nutrients (TC and TN) caused by soil salinization indirectly increased the number of animal pathogens. These reports were further supported by Chen et al. (2019), who showed that the relative abundance of animal pathogens was negatively associated with nutrition substrates.

### Key Properties Affecting Soil Fungal Communities and Functions

Soil fungal communities are significantly affected by environmental factors (Leff et al., 2015; Bachelot et al., 2016). Our previous study suggested that the best indicator of soil structure quality (Pagliai and Vignozzi, 2002), soil total porosity (calculated from soil BD), can influence soil fungal communities (Yang et al., 2019). Soil salinization can significantly increase soil BD, reduce soil porosity, and indirectly regulate soil microbial structure (Zhao et al., 2017). We observed a stronger positive correlation between soil salinity and soil BD, indicating that salinization changes soil from an aerobic environment (more oxygen) to anaerobic environment (less oxygen). Under aerobic conditions, *Nectriaceae* and *Plectosphaerellaceae* were characterized by high abundance, which indicated a high demand for oxygen; however, anaerobic conditions were conducive to the growth of *Cladosporiaceae*, *Bulleribasidiaceae*, and *Aspergillaceae*. In addition to soil BD, many studies have implied that soil pH is one of the most important factors affecting soil fungal communities (Maestre et al., 2015; Hu et al., 2017). Zhao et al. (2018) suggested that pH is an equally important environmental factor controlling the bacterial community structure as salinity in northwestern China. In saline-alkali soils of the temperate grasslands in northeastern China, our previous findings suggested that soil pH was negatively correlated with fungal diversity compared with soil salinity and the C/N ratio (Yang et al., 2020). However, in the present study, soil salinity (EC and salt content) had a stronger effect on the soil fungal communities than the soil pH and soil TC according to the Mantel tests test and the Pearson correlation analyses. Results obtained from 16S rRNA high-throughput sequencing further strengthened our observations in the YRD. Zhao et al. (2020) reported a slight influence of pH on bacterial community compositions and diversities. Soil pH showed a significant correlation only with the abundance of *Cytophagia*. Our observations are in line with the above, where soil pH significantly negatively correlated with only the relative abundances of *Plectosphaerellaceae*. We speculate that soil microorganisms in northeastern China are mainly affected by soil pH (Li and Yang, 2019; Yang et al., 2020), compared with soil pH and salinity in northwestern China (Zhao et al., 2018), and salinity (soil EC and salt content) in the YRD (Zhao et al., 2020).

Different fungi with different ecological functions had different relationships with soil properties, and Schmidt et al. (2019) reported that the relative proportion of arbuscular mycorrhizae was negatively correlated with soil pH (neutral soil) in agroecosystems; however, in this study, soil pH (highly alkaline soil) had no significant correlation with the relative proportion of arbuscular mycorrhizae, indicating that different range of pH values had different effects on soil arbuscular mycorrhizae. A study by Dighton (2003) showed that the soil carbon decomposition rates were primarily regulated by fungal saprotrophs, and the relative increase in saprotrophs was associated with increased nutrient content (Schmidt et al., 2019). Our results showed that the relative proportion of litter saprotrophs was positively correlated with soil C and N concentrations in salinity soils of YRD. In particular, the gradient of saline alkali in the experimental plot increased unevenly, and the soil salt content in low and medium saline soils showed no significant difference. In this way, animal pathogens showed no significant difference among gradients, but they were significantly positively correlated with soil salt content. Our results suggest that soil salinity decreased the abundance of litter saprotrophs and increased the abundance of animal pathogens, which increased our understanding of the impact of salinization on soil health. We recommend further investigation into the ecosystem functions of soil fungi in the extremely saline-alkaline soils.

## Conclusion

Our study explored the distribution patterns of soil fungal communities and diversities in the extremely saline-alkaline soils of the YRD. The soil salt, EC, and BD values significantly increased, while significantly lower values of soil TC and TN were observed as salinization increased. Significantly lower values of the Shannon and Chao indexes were observed in extremely saline soil. Additionally, the CH index showed that the data naturally separated into two clusters based on the JSD method, and the relatively high levels of the families *Nectriaceae* and *Cladosporiaceae* distinguished two of the clusters, indicating two enterotypes of low and high salinity soils, respectively. The *Nectriaceae* and *Cladosporiaceae* were the families most positively and negatively correlated with others, respectively, based on the network analysis. Plant saprotrophs and litter saprotrophs were significantly lower in extremely saline soil than in non-saline soil. Our results suggest that soil salinity is a primary factor that shapes soil fungal communities and provides a framework for future research to deeply analyze the mechanism and function of salt tolerance of soil fungi in saline-alkaline environments.

## Data Availability Statement

The datasets presented in this study can be found in online repositories. The names of the repository/repositories and accession number(s) can be found at: https://www.ncbi.nlm.nih.gov/, SRP269059.

## Author Contributions

CY and JS designed the study. CY participated in sample collection, performed the experiment, and wrote the manuscript with the help of JS. All authors contributed to the article and approved the submitted version.

### Conflict of Interest

The authors declare that the research was conducted in the absence of any commercial or financial relationships that could be construed as a potential conflict of interest.

## References

[ref1] Auguet J. -C. Barberan A. Casamayor E. O. (2010). Global ecological patterns in uncultured archaea. ISME J. 4, 182–190. 10.1038/ismej.2009.109, PMID: 19847207

[ref2] Bachelot B. Uriarte M. Zimerman J. K. Thompson J. Leff J. W. Asiaii A.. (2016). Long-lasting effects of land use history on soil fungal communities in second-growth tropical rain forests. Ecol. Appl. 26, 1881–1895. 10.1890/15-1397.1, PMID: 27755697

[ref3] Campbell B. J. Kirchman D. L. (2013). Bacterial diversity, community structure and potential growth rates along an estuarine salinity gradient. ISME J. 7, 210–220. 10.1038/ismej.2012.93, PMID: 22895159 PMC3526181

[ref4] Chen J. Xu H. He D. Li Y. Luo T. Yang H. (2019). Historical logging alters soil fungal community composition and network in a tropical rainforest. Forest Ecol. Manag. 433, 228–239. 10.1016/j.foreco.2018.11.005

[ref5] Chowdhury N. Marschner P. Burns R. (2011). Response of microbial activity and community structure to decreasing soil osmotic and matric potential. Plant Soil 344, 241–254. 10.1007/s11104-011-0743-9

[ref6] Crowther T. W. Boddy L. Jones T. H. (2012). Functional and ecological consequences of saprotrophic fungus-grazer interactions. ISME J. 6, 1992–2001. 10.1038/ismej.2012.53, PMID: 22717883 PMC3475375

[ref7] Cubasch U. Wuebbles D. Chen D. Facchini M. C. Frame D. Mahowald N. (2014). Climate change 2013: The physical science basis working group I contribution to the fifth assessment report of the intergovernmental panel on climate change introduction.

[ref8] Cui B. Yang Q. Yang Z. Zhang K. (2009). Evaluating the ecological performance of wetland restoration in the yellow river delta, China. Ecol. Eng. 35, 1090–1103. 10.1016/j.ecoleng.2009.03.022

[ref9] Dighton J. (2003). “Fungi in ecosystem processes (mycology 17)” in Fungi and primary productivity: Making nutrients available. ed. Bennett J. W. (New York, USA: Marcel Dekker Inc.).

[ref10] Elmajdoub B. Marschner P. (2015). Responses of soil microbial activity and biomass to salinity after repeated additions of plant residues. Pedosphere 25, 177–185. 10.1016/S1002-0160(15)60002-9

[ref11] Geml J. Pastor N. Fernandez L. Pacheco S. Semenova T. A. Becerra A. G.. (2014). Large-scale fungal diversity assessment in the Andean Yungas forests reveals strong community turnover among forest types along an altitudinal gradient. Mol. Ecol. 23, 2452–2472. 10.1111/mec.12765, PMID: 24762095

[ref12] Hu X. Liu J. Wei D. Zhu P. Cui X. A. Zhou B. (2017). Effects of over 30-year of different fertilization regimes on fungal community compositions in the black soils of Northeast China. Agric. Ecosyst. Environ. 248, 113–122. 10.1016/j.agee.2017.07.031

[ref13] Hu Y. Wang L. Xi X. Hu J. Hou Y. Le Y. (2016). Effects of salinity on soil bacterial and archaeal community in estuarine wetlands and its implications for carbon sequestration: verification in the yellow river delta. Chem. Ecol. 32, 669–683. 10.1080/02757540.2016.1177519

[ref14] Kim K. Samaddar S. Chatterjee P. Krishnamoorthy R. Jeon S. Sa T. (2019). Structural and functional responses of microbial community with respect to salinity levels in a coastal reclamation land. Appl. Soil Ecol. 137, 96–105. 10.1016/j.apsoil.2019.02.011

[ref15] Kramer S. Marhan S. Ruess L. Armbruster W. Butenschoen O. Haslwimmer H. (2012). Carbon flow into microbial and fungal biomass as a basis for the belowground food web of agroecosystems. Pedobiologia 55, 111–119. 10.1016/j.pedobi.2011.12.001

[ref16] Krishnamoorthy R. Kim K. Kim C. Sa T. (2014). Changes of arbuscular mycorrhizal traits and community structure with respect to soil salinity in a coastal reclamation land. Soil Biol. Biochem. 72, 1–10. 10.1016/j.soilbio.2014.01.017

[ref17] Leff J. W. Jones S. E. Prober S. M. Barberan A. Borer E. T. Firn J. L.. (2015). Consistent responses of soil microbial communities to elevated nutrient inputs in grasslands across the globe. Proc. Natl. Acad. Sci. U. S. A. 112, 10967–10972. 10.1073/pnas.1508382112, PMID: 26283343 PMC4568213

[ref18] Li J. J. Yang C. (2019). Inconsistent response of soil bacterial and fungal communities in aggregates to litter decomposition during short-term incubation. PeerJ 7:e8078. 10.7717/peerj.8078, PMID: 31741807 PMC6858812

[ref19] Lozupone C. A. Knight R. (2007). Global patterns in bacterial diversity. Proc. Natl. Acad. Sci. U. S. A. 104, 11436–11440. 10.1073/pnas.0611525104, PMID: 17592124 PMC2040916

[ref20] Maestre F. T. Delgado-Baquerizo M. Jeffries T. C. Eldridge D. J. Ochoa V. Gozalo B.. (2015). Increasing aridity reduces soil microbial diversity and abundance in global drylands. Proc. Natl. Acad. Sci. U. S. A. 112, 15684–15689. 10.1073/pnas.1516684112, PMID: 26647180 PMC4697385

[ref21] Mohamed D. J. Martiny J. B. (2011). Patterns of fungal diversity and composition along a salinity gradient. ISME J. 5, 379–388. 10.1038/ismej.2010.137, PMID: 20882058 PMC3105720

[ref22] Morrissey E. M. Gillespie J. L. Morina J. C. Franklin R. B. (2014). Salinity affects microbial activity and soil organic matter content in tidal wetlands. Glob. Chang. Biol. 20, 1351–1362. 10.1111/gcb.12431, PMID: 24307658

[ref23] Nguyen N. H. Song Z. Bates S. T. Branco S. Tedersoo L. Menke J. (2016). FUNGuild: an open annotation tool for parsing fungal community datasets by ecological guild. Fungal Ecol. 20, 241–248. 10.1016/j.funeco.2015.06.006

[ref24] Pagliai M. Vignozzi N. (2002). The soil pore system as an indicator of soil quality. Adv. Geoecol. 35, 71–82.

[ref25] Rath K. M. Fierer N. Murphy D. V. Rousk J. (2019a). Linking bacterial community composition to soil salinity along environmental gradients. ISME J. 13, 836–846. 10.1038/s41396-018-0313-8, PMID: 30446737 PMC6461869

[ref26] Rath K. M. Maheshwari A. Rousk J. (2019b). Linking microbial community structure to trait distributions and functions using salinity as an environmental filter. mBio 10:e01607–19. 10.1128/mBio.01607-19, PMID: 31337729 PMC6650560

[ref27] Rath K. M. Rousk J. (2015). Salt effects on the soil microbial decomposer community and their role in organic carbon cycling: a review. Soil Biol. Biochem. 81, 108–123. 10.1016/j.soilbio.2014.11.001

[ref28] Schmidt R. Mitchell J. Scow K. (2019). Cover cropping and no-till increase diversity and symbiotroph:saprotroph ratios of soil fungal communities. Soil Biol. Biochem. 129, 99–109. 10.1016/j.soilbio.2018.11.010

[ref29] Takami H. (2011). “Genomics and evolution of alkaliphilic bacillus species” in Extremophiles handbook. ed. Horikoshi K. (Tokyo: Springer Japan), 183–211.

[ref30] Tyakht A. V. Kostryukova E. S. Popenko A. S. Belenikin M. S. Pavlenko A. V. Larin A. K.. (2013). Human gut microbiota community structures in urban and rural populations in Russia. Nat. Commun. 4:2469. 10.1038/ncomms3469, PMID: 24036685 PMC3778515

[ref31] van der Heijden M. G. A. Bardgett R. D. van Straalen N. M. (2008). The unseen majority: soil microbes as drivers of plant diversity and productivity in terrestrial ecosystems. Ecol. Lett. 11, 296–310. 10.1111/j.1461-0248.2007.01139.x, PMID: 18047587

[ref32] van der Wal A. Geydan T. D. Kuyper T. W. de Boer W. (2013). A thready affair: linking fungal diversity and community dynamics to terrestrial decomposition processes. FEMS Microbiol. Rev. 37, 477–494. 10.1111/1574-6976.12001, PMID: 22978352

[ref33] Vega F. E. Goettel M. S. Blackwell M. Chandler D. Jackson M. A. Keller S. (2009). Fungal entomopathogens: new insights on their ecology. Fungal Ecol. 2, 149–159. 10.1016/j.funeco.2009.05.001

[ref34] Wang M. Chen S. Chen L. Wang D. (2019). Responses of soil microbial communities and their network interactions to saline-alkaline stress in cd-contaminated soils. Environ. Pollut. 252, 1609–1621. 10.1016/j.envpol.2019.06.082, PMID: 31284203

[ref35] Wang C. Wang S. (2017). “Insect pathogenic fungi: genomics, molecular interactions, and genetic improvements” in Annual review of entomology. Vol. 62 ed. Berenbaum M. R. (USA: Palo Alto), 73–90. 10.1146/annurev-ento-031616-035509 27860524

[ref36] White T. J. Bruns T. Lee S. Taylor J. (1990). “Amplification and direct sequencing of fungal ribosomal RNA genes for phylogenetics” in PCR protocols: A guide to methods and applications. eds. Innis M. A., D. H. Gelfand, J. J. Sninsky and T. J. White (California, USA and London, England, UK: Academic Press, Inc.), 315–322.

[ref37] Wong V. N. L. Greene R. S. B. Dalal R. C. Murphy B. W. (2010). Soil carbon dynamics in saline and sodic soils: a review. Soil Use Manag. 26, 2–11. 10.1111/j.1475-2743.2009.00251.x

[ref38] Yang C. Liu N. Zhang Y. (2019). Soil aggregates regulate the impact of soil bacterial and fungal communities on soil respiration. Geoderma 337, 444–452. 10.1016/j.geoderma.2018.10.002

[ref39] Yang C. Wang X. Miao F. Li Z. Tang W. Sun J. (2020). Assessing the effect of soil salinization on soil microbial respiration and diversities under incubation conditions. Appl. Soil Ecol. 155:103671. 10.1016/j.apsoil.2020.103671

[ref40] Yang W. Zhao H. Leng X. Cheng X. An S. (2017). Soil organic carbon and nitrogen dynamics following *Spartina alterniflora* invasion in a coastal wetland of eastern China. Catena 156, 281–289. 10.1016/j.catena.2017.03.021

[ref41] Zhao Q. Bai J. Gao Y. Zhao H. Zhang G. Cui B. (2020). Shifts in the soil bacterial community along a salinity gradient in the yellow river delta. Land Degrad. Dev. 31, 2255–2267. 10.1002/ldr.3594

[ref42] Zhao Q. Bai J. Lu Q. Zhang G. (2017). Effects of salinity on dynamics of soil carbon in degraded coastal wetlands: implications on wetland restoration. Phys. Chem. Earth 97, 12–18. 10.1016/j.pce.2016.08.008

[ref43] Zhao S. Liu J. J. Banerjee S. Zhou N. Zhao Z. Y. Zhang K.. (2018). Soil pH is equally important as salinity in shaping bacterial communities in saline soils under halophytic vegetation. Sci. Rep. 8:4550. 10.1038/s41598-018-22788-7, PMID: 29540760 PMC5851986

[ref44] Zheng W. Xue D. Li X. Deng Y. Rui J. Feng K. (2017). The responses and adaptations of microbial communities to salinity in farmland soils: a molecular ecological network analysis. Appl. Soil Ecol. 120, 239–246. 10.1016/j.apsoil.2017.08.019

[ref45] Zhou J. Deng Y. Luo F. He Z. Yang Y. (2011). Phylogenetic molecular ecological network of soil microbial communities in response to elevated CO_2_. mBio 2:e00122–11. 10.1128/mBio.00122-11, PMID: 21791581 PMC3143843

