# Supplementary material for: Soil Salinity Drives the Distribution Patterns and Ecological Functions of Fungi in Saline-Alkali Land in the Yellow River Delta, China
Source: Front Microbiol. 2020 Dec 23;11:594284. doi: 10.3389/fmicb.2020.594284 (PMC7786015; doi:10.3389/fmicb.2020.594284)
Supplement: Supplementary file 1 [file Data_Sheet_1.docx]

Supplementary Material

# Supplementary Figures and Tables


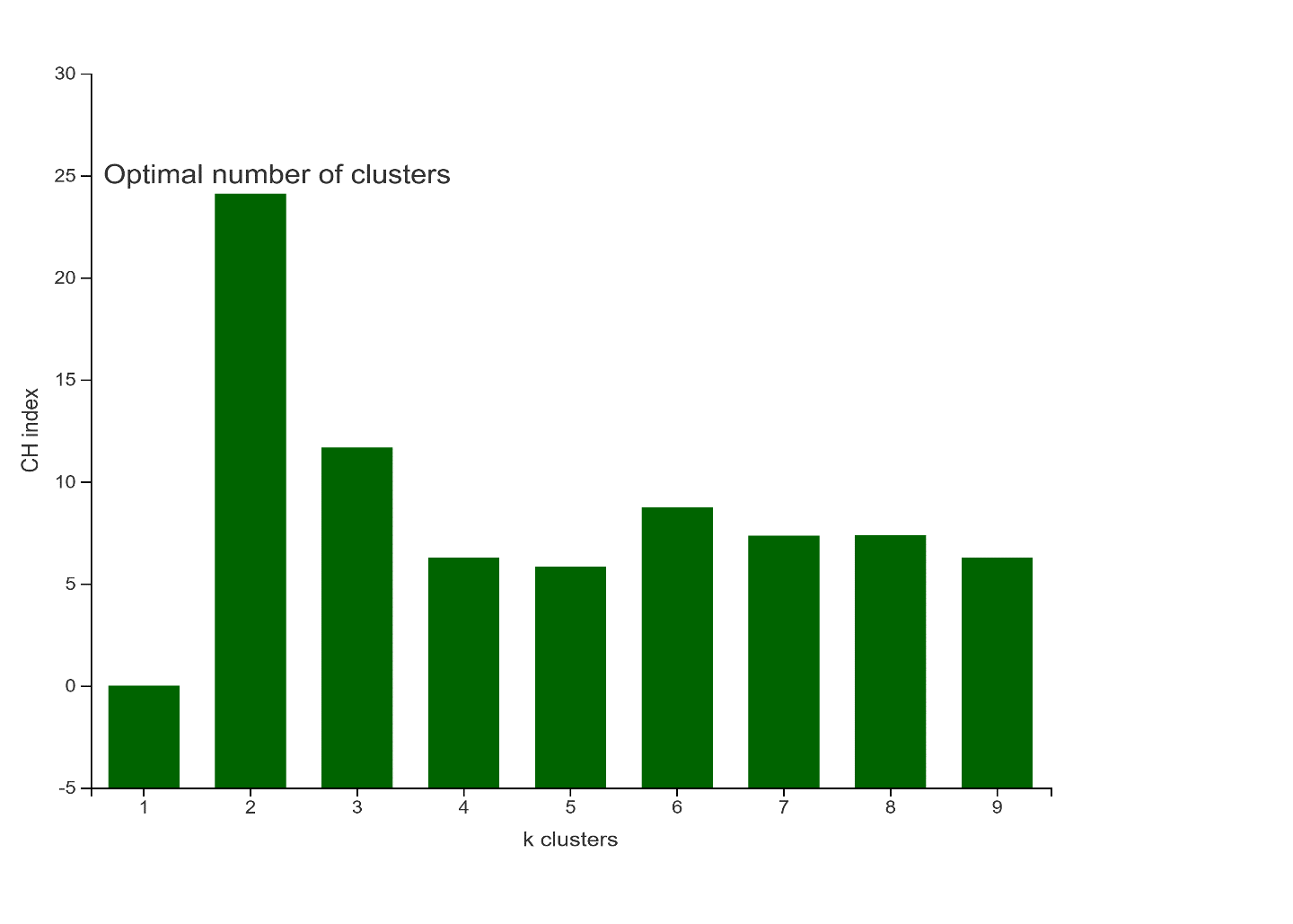


**Fig. S1** Calinski–Harabasz (CH) index shows that the data are most naturally separated into two clusters using Jensen-Shannon distance method.

**Table S1** Network node attribute table.

| **node name** | **degree** | **clustering** |
| --- | --- | --- |
| f__Nectriaceae | 22 | 0.467532468 |
| f__unclassified_o__Chaetothyriales | 20 | 0.584210526 |
| f__unclassified_c__Sordariomycetes | 20 | 0.584210526 |
| f__Plectosphaerellaceae | 20 | 0.568421053 |
| f__unclassified_o__GS11 | 18 | 0.666666667 |
| f__unclassified_o__Hypocreales | 17 | 0.617647059 |
| f__Cladosporiaceae | 17 | 0.588235294 |
| f__Mortierellaceae | 16 | 0.566666667 |
| f__unclassified_o__Branch06 | 15 | 0.495238095 |
| f__Trichosporonaceae | 15 | 0.542857143 |
| f__Filobasidiaceae | 15 | 0.619047619 |
| f__Aspergillaceae | 14 | 0.571428571 |
| f__Myrmecridiaceae | 14 | 0.659340659 |
| f__Leptosphaeriaceae | 14 | 0.747252747 |
| f__Ceratobasidiaceae | 14 | 0.714285714 |
| f__unclassified_k__Fungi | 13 | 0.602564103 |
| f__Psathyrellaceae | 11 | 0.727272727 |
| f__Hypocreaceae | 10 | 0.555555556 |
| f__unclassified_o__Olpidiales | 10 | 0.533333333 |
| f__Sporidiobolaceae | 9 | 0.305555556 |
| f__Lasiosphaeriaceae | 9 | 0.527777778 |
| f__Trichocomaceae | 8 | 0.392857143 |
| f__Bionectriaceae | 8 | 0.428571429 |
| f__Glomeraceae | 7 | 0.571428571 |
| f__unclassified_o__Tremellales | 7 | 0.285714286 |
| f__Wallemiaceae | 7 | 0.285714286 |
| f__Sporormiaceae | 7 | 0.380952381 |
| f__Microascaceae | 6 | 0.266666667 |
| f__Clavicipitaceae | 6 | 0.266666667 |
| f__Lulworthiaceae | 5 | 0.6 |
| f__Pleosporaceae | 5 | 0.6 |
| f__Stachybotryaceae | 5 | 0.2 |
| f__Phaeosphaeriaceae | 5 | 0.4 |
| f__Chaetomiaceae | 5 | 0.2 |
| f__Cordycipitaceae | 5 | 0.6 |
| f__Didymosphaeriaceae | 5 | 0.5 |
| f__unclassified_o__Sordariales | 5 | 0.5 |
| f__Bulleribasidiaceae | 4 | 0.666666667 |
| f__unclassified_o__Microascales | 4 | 0.166666667 |
| f__Mycosphaerellaceae | 3 | 0.666666667 |
| f__unclassified_p__Ascomycota | 3 | 0.666666667 |
| f__unclassified_o__Pleosporales | 2 | 0 |
| f__Hypocreales_fam_Incertae_sedis | 2 | 1 |
| f__Pseudeurotiaceae | 2 | 1 |
| f__Diatrypaceae | 2 | 0 |
| f__Didymellaceae | 1 | 0 |
| f__Symmetrosporaceae | 1 | 0 |
| f__Rhynchogastremataceae | 1 | 0 |

Note: Degree represents the number of nodes connected to a node; clustering is coefficient of a node, indicating the connection between a node and its adjacent nodes: if a node is completely connected with its adjacent nodes, the clustering coefficient is 1; if a node has almost no connection with its adjacent nodes, the clustering coefficient is close to 0, and the clustering coefficient is larger indicates that the node is more important.

**Table S2** Partial Mantel tests (permutation = 999) between soil properties (pH, EC, BD, salt, TC, TN, and C/N ratio) and the top ten fungi in abundance, and the resulting significance level was tested by the Mantel r statistic and p values.

| Partial Mantel tests | Mantel r | *p*-value |
| --- | --- | --- |
| EC | **0.61** | **0.001** |
| Salt | **0.48** | **0.001** |
| BD | **0.54** | **0.001** |
| pH | **0.21** | **0.021** |
| TC | **0.55** | **0.001** |
| TN | 0.65 | 0.001 |
| C/N ratio | 0.52 | 0.001 |
